# Bilateral Diabetic Knee Neuroarthropathy in a Forty-Year-Old Patient

**DOI:** 10.1155/2016/3204813

**Published:** 2016-09-07

**Authors:** Patrick Goetti, Nicolas Gallusser, Olivier Borens

**Affiliations:** Department of Orthopedics and Traumatology, Lausanne University Hospital, rue du Bugnon 46, 1011 Lausanne, Switzerland

## Abstract

Diabetic osteoarthropathy is a rare cause of neuropathic joint disease of the knee; bilateral involvement is even more exceptional. Diagnosis is often made late due to its unspecific symptoms and appropriate surgical management still needs to be defined, due to lack of evidence because of the disease's low incidence. We report the case of a forty-year-old woman with history of diabetes type I who developed bilateral destructive Charcot knee arthropathy. Bilateral total knee arthroplasty was performed in order to achieve maximal functional outcome. Follow-up was marked by bilateral tibial periprosthetic fractures treated by osteosynthesis with a satisfactory outcome. The diagnosis of Charcot arthropathy should always be in mind when dealing with atraumatic joint destruction in diabetic patients. Arthroplasty should be considered as an alternative to arthrodesis in bilateral involvement in young patients.

## 1. Introduction

The first anatomopathological description of neuropathic joint destruction was reported by Jean-Martin Charcot in 1868. While many disorders have been related to neuropathic joint arthropathy, diabetes mellitus is nowadays the primary etiology [1–4]. The prevalence of diabetic osteoarthropathy lies between 0.1 and 13% [5]. Bilateral Charcot arthropathy is a rare condition [6]. The diagnosis is often made late due to the unspecific early presentation of brutal inflammatory joint pain, which can also be misdiagnosed as common fracture, infectious, rheumatic arthritis, deep venous thrombosis, algoneurodystrophy, or erysipelas [7]. The diagnosis is made with standard X-rays and inflammatory parameters on blood tests. Due to its low incidence, the appropriate surgical treatment is still controversial with a trend going towards total knee arthroplasty (TKA).

## 2. Case Presentation

**Figure 1:**
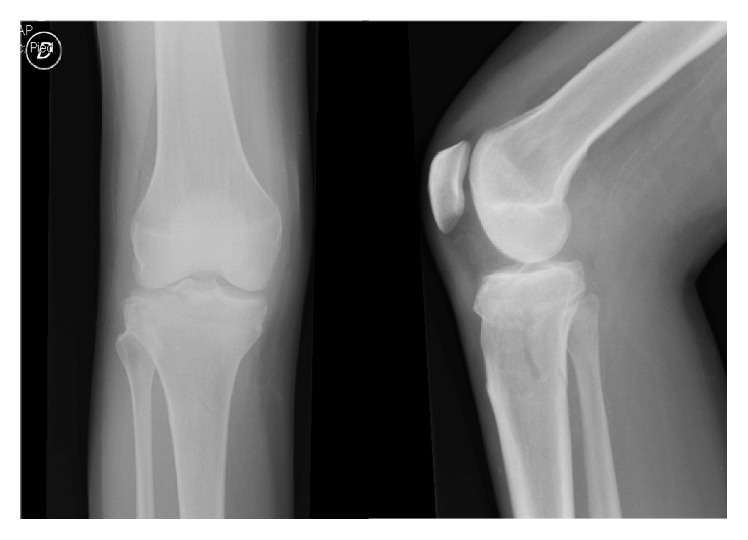
Initial X-rays of the right knee.

**Figure 2:**
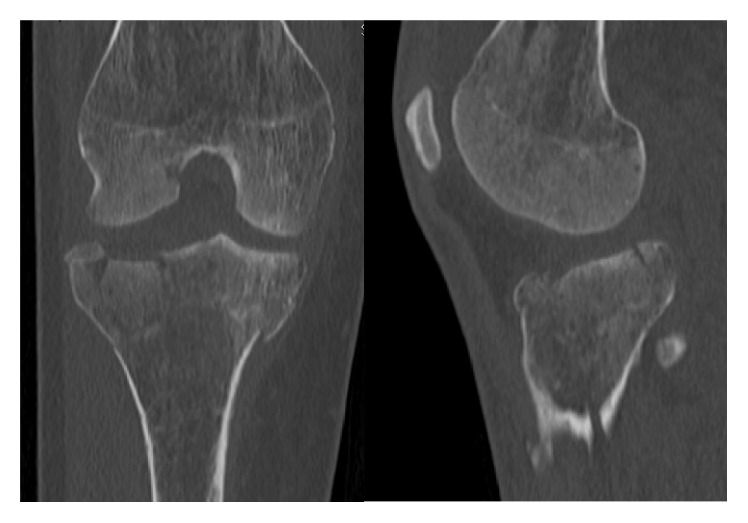
Initial CT-scan of the right knee.

**Figure 3:**
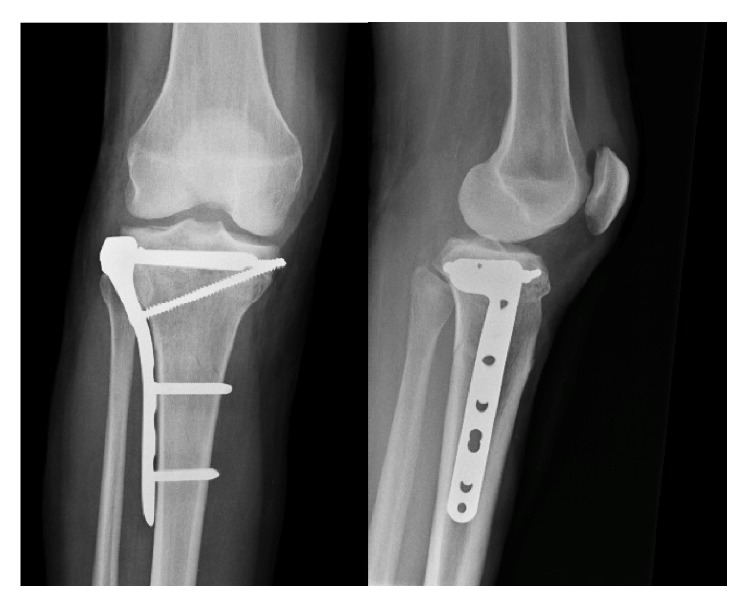
X-rays of the right knee after osteosynthesis.

**Figure 4:**
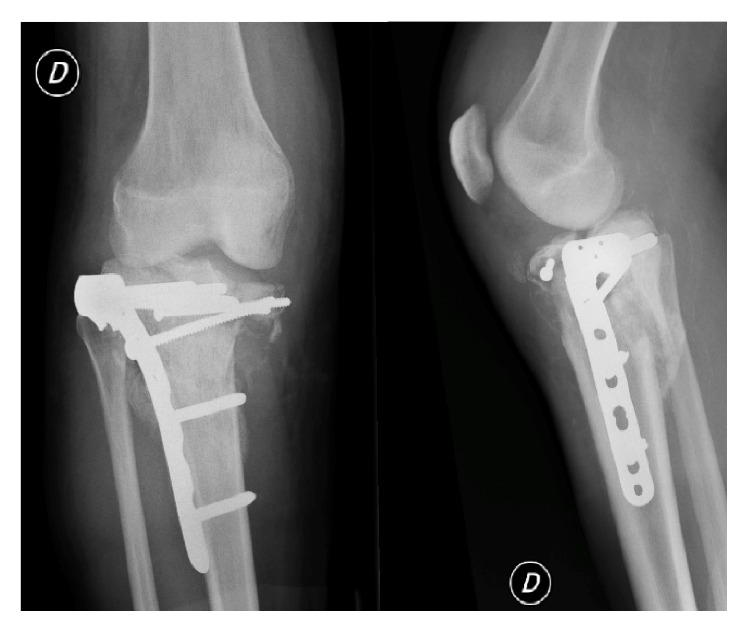
Follow-up X-rays at three months postoperatively of the right knee.

**Figure 5:**
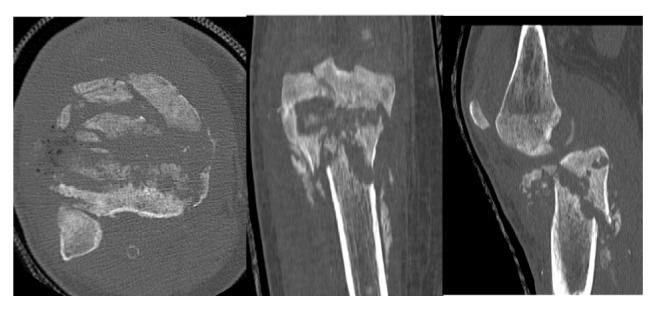
CT-scan after plate removal of the right knee.

A forty-year-old woman with history of type I diabetes mellitus complicated with diabetic neuropathy and Charcot disease of her right foot is referred from her general practitioner with right knee pain without history of trauma. The initial X-rays revealed a Schatzker type V tibial plateau fracture which was surgically treated by open reduction and lateral plate osteosynthesis using a locking compression plate (LCP) (Figures 1, 2, and 3). Progressive secondary fracture displacement on follow-up X-rays associated with necrosis of the medial tibial plateau and finally plate failure at three months postoperatively was observed (Figure 4). Varus pseudolaxity was present on clinical examination, a low-grade infection was suspected, and removal of hardware and wide debridement were performed. A postoperative CT-scan showed complete articular destruction of the medial and lateral tibial plateau (Figure 5). Sonication of the implant and standard microbiological exams remained negative. Due to the important destruction of the proximal tibia, a cemented rotating hinged knee prosthesis (RHK, Zimmer®) was implanted. Postoperatively the patient stayed for six weeks with partial weight bearing. Knee range of motion (ROM) was 105/0/0° (flexion/extension/hyperextension) at nine days postoperatively. Wound healing was uneventful in a satisfied patient with no complaints about her knee.

**Figure 6:**
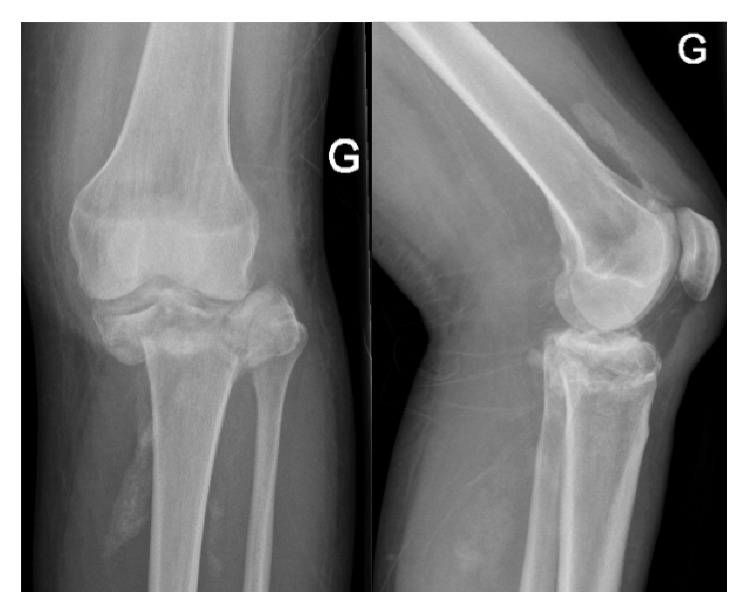
Initial X-rays of the left knee.

**Figure 7:**
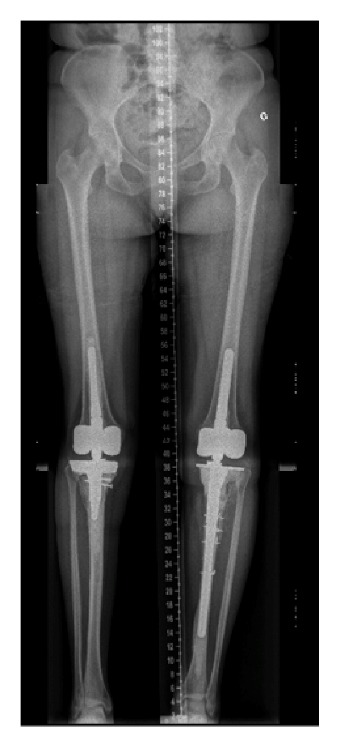
Follow-up X-rays, three months after osteosynthesis of the left tibial periprosthetic fracture.

Eighteen months after initial management of the right knee, the patient presented at our outpatients' clinic with complaints of progressive invalidating contralateral knee pain. Performing the X-rays of the left knee, we were suspicious of a Charcot arthropathy (Figure 6). None-weight bearing immobilization of the left limb was initiated and one month later a cemented Zimmer® RHK prosthesis was implanted. Reeducation was performed with partial weight bearing of the left limb and knee ROM was 90/0/0° at seven days postoperatively. At one month postoperatively she started complaining of increasing left diaphyseal tibial pain without trauma. X-rays revealed a periprosthetic fracture around the tibial shaft. The patient was taken back to the operating room (OR) for revision surgery with replacement of the tibial stem and cerclage of the tibial shaft fracture. At three months postoperatively the patient still needed two crutches to walk. The scars were calm, and knee ROM was 100/0/3° on the left side and 120/0/5° on the right side (Figure 7).

At ten months postoperatively the patient was readdressed with left tibial pain. X-rays revealed a displaced fracture below the tip of the revision tibial stem, which had appeared at seven months postoperatively and had been initially treated with cast immobilization and none-weight bearing at an outside hospital. The patient was taken back to the OR and LCP plating of the distal left tibia and peroneus was performed.

At one year postoperatively the patient was addressed to emergency department with a periprosthetic fracture of the proximal right tibia without any trauma. Revision surgery with replacement of the tibial implant by a long stem bridging the fracture line was performed. At six weeks postoperatively the patient was able to walk with two crutches and presented good wound healing. Knee ROM was 95/0/0° on both sides.

**Figure 8:**
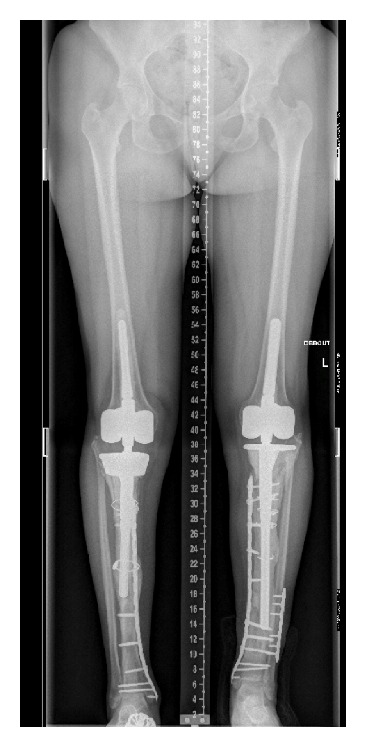
Follow-up X-rays, twelve months after osteosynthesis of the right tibial periprosthetic fracture.

She was finally seen again two months later after a fall down the stairs with a periprosthetic fracture of the distal tibia. The fracture was dealt with by LCP plating of the right distal tibia. After consultation of our Rheumatology Department, a six-month off-label prescription of teriparatide (Forsteo®) was introduced to promote bone healing and prevent further fractures. Follow-up was uneventful at one year postoperatively. She walked with two crutches and knee ROM was 95/0/0° on both sides (Figure 8).

## 3. Discussion

Neuropathic arthropathy is a rare complication of diabetes mellitus, which can occur in one or more joints. Knee involvement however is exceptional [5]. Standard treatment implies long term glycemic control to obtain disease stabilization and none-weight bearing cast immobilization [7]. Nevertheless, this treatment is difficult to apply to the knee especially in the case of a young and active patient.

Medical treatment with alendronate and calcitonin has been proposed, but there is at the moment not enough evidence in the literature to recommend their systematic clinical use [8]. In our case we introduced teriparatide at the end of the surgical management. To our knowledge, there is only one case report of a successful use of teriparatide in ankle Charcot arthropathy [9]; we think there is a need for further studies to confirm this indication. The gold standard for surgical treatment used to be arthrodesis due to the high complication rates of TKR [10, 11]. With recent improvement in long term survival of total knee replacement in neuropathic joint destruction with 85% survival at 8 years as reported by Parvizi [12, 13], TKR is nowadays a valid option with a higher functional outcome than arthrodesis by conserving knee joint function and with fewer issues on leg length discrepancy.

Neuropathic arthropathy can be classified in three stages using standard radiology (developmental, coalescence, and reconstructive) using the Eichenholtz classification. A better outcome is obtained if implantation occurs after the initial developmental phase [14]. In our case, total knee arthroplasty was performed rather early.

We chose a cemented fixation to achieve a good primary fixation and an RHK design was used because of the preexisting deformity and associated ligamentous insufficiency. One described complication of early implantation is aseptic loosening due to the increased stress on the bone cement interface, which was not present in our case [15]. We were on the other hand confronted with a high complication rate with bilateral periprosthetic fractures. We refer them to the stress riser induced by the modulus mismatch between the stem and the patient's extremely narrow tibial diaphysis.

## 4. Conclusion

The diagnosis of Charcot arthropathy should always be in mind when dealing with atraumatic joint destruction in diabetic patients. There is still a lack of consensus regarding the optimal treatment when operative management is indicated. In our opinion total knee arthroplasty (TKA) should be preferred over arthrodesis in young patients. Nonetheless, TKA for neuropathic arthropathy is associated with high complication rates. It is technically very demanding and often needs operative techniques and implants otherwise reserved for complex revision arthroplasties.
